# What Is—and What Is Not—Immunogenic Cell Death? Functional Definitions, Experimental Standards, and Common Pitfalls

**DOI:** 10.3390/ijms27073061

**Published:** 2026-03-27

**Authors:** Diego Liviu Boaru, Oscar Fraile-Martinez, Patricia De Castro-Martinez, Miguel A. Ortega, Cielo Garcia-Montero

**Affiliations:** 1 Department of Medicine and Medical Specialties, Centro de Investigación Biomédica en Red en el Área Temática de Enfermedades Hepáticas (CIBEREHD), Faculty of Medicine and Health Sciences, University of Alcalá, 28801 Alcala de Henares, Spain; diego.boaru@edu.uah.es (D.L.B.); oscar.fraile@uah.es (O.F.-M.); patriciadecastro1999@gmail.com (P.D.C.-M.); cielo.gmontero@gmail.com (C.G.-M.); 2 Ramón y Cajal Institute of Sanitary Research (IRYCIS), 28034 Madrid, Spain

**Keywords:** immunogenic cell death, damage-associated molecular patterns (DAMPs), dendritic cells, antigen presentation, CD8^+^ T cells, tumor microenvironment

## Abstract

Immunogenic cell death (ICD) links tumor cell demise to the activation of anti-tumor immunity, but its adoption has also generated inconsistent definitions and frequent overinterpretation of surrogate biomarkers. Here, we synthesize mechanistic and methodological evidence showing that danger-associated molecular patterns (DAMPs), cytokine release, and endoplasmic reticulum stress report immunogenic potential rather than ICD itself. We propose that ICD should be defined by its functional immunological endpoint, namely efficient antigen presentation and antigen-specific adaptive immunity, ideally culminating in protective immunological memory. To operationalize this principle, we introduce a hierarchy of experimental validation ranging from correlative hallmarks (Level 0) to innate immune integration (Level 1), antigen-specific T-cell priming (Level 2), definitive vaccination-rechallenge protection with immune-dependence testing (Level 3), and translational relevance supported by convergent human data (Level 4). We also discuss common pitfalls, equating inflammation, necrosis-associated DAMP release, or therapeutic benefit with ICD, and outline minimal immune-context controls (e.g., MHC-I, CD8^+^ T cells, Batf3-dependent dendritic cells, and innate sensing pathways) required to support robust claims. Finally, we highlight why ICD remains strongly context-dependent, shaped by dendritic-cell competence, innate licensing, purinergic metabolism, and microenvironmental constraints. Evidence-graded standards should improve reproducibility, strengthen peer review, and accelerate clinically meaningful ICD-based strategies.

## 1. Introduction

Cell death is a fundamental biological process required for tissue homeostasis, development, and adaptation to stress [1]. In cancer, however, the deregulation of cell death pathways profoundly shapes tumor progression and therapeutic responses [2]. Beyond the classical distinction between apoptotic and non-apoptotic forms of regulated cell death (RCD), it has become increasingly evident that not all dying cells are perceived equally by the immune system [3]. While many forms of cell death are immunologically silent, or even actively tolerogenic, others can stimulate robust innate and adaptive immune responses [4]. This latter category, termed immunogenic cell death (ICD), has emerged as a key conceptual bridge between cancer cell-intrinsic stress responses and anti-tumor immunity.

ICD was originally described in the mid-2000s as a form of regulated cell death capable of converting dying tumor cells into a source of antigens and adjuvant signals that alert the immune system [5]. ICD is characterized by the emission of danger signals that promote immune recognition. These signals include the extracellular release or surface exposure of damage-associated molecular patterns (DAMPs), which act as immunostimulatory cues for antigen-presenting cells (APCs), particularly dendritic cells (DCs) [6,7]. Through this process, dying cancer cells can initiate a cascade of events that culminate in tumor antigen presentation, priming of cytotoxic T lymphocytes (CTLs), and the establishment of long-lasting anti-tumor immune memory [8].

Among the most extensively studied DAMPs associated with ICD are calreticulin (CALR), adenosine triphosphate (ATP), high-mobility group box 1 (HMGB1), and heat shock proteins such as HSP70 and HSP90 [9,10,11]. These molecules are normally confined to intracellular compartments, where they perform essential housekeeping functions. During ICD, however, they are either exposed on the cell surface or released into the extracellular milieu, where they acquire immunomodulatory roles [12]. Surface-exposed CALR functions as an “eat-me” signal that promotes phagocytosis of dying cells by APCs, whereas extracellular ATP and HMGB1 act as chemoattractants and activation signals that support DC recruitment, maturation, and antigen processing [13,14]. Together, these constitutively expressed DAMPs facilitate the uptake and cross-presentation of tumor antigens to CD8^+^ T cells, thereby linking cell death to adaptive immunity.

In addition to these pre-existing signals, ICD can also induce the transcriptional activation of cytokines and chemokines, often referred to as inducible DAMPs, including type I interferons, interleukin-1 family members, and chemokines such as CXCL10 [15]. These mediators amplify immune cell recruitment and function through pathways involving pattern recognition receptors, inflammasomes, and interferon regulatory factors [16]. Endogenous nucleic acids released from dying cells, as well as exogenous pathogen-associated molecular patterns in the context of oncolytic virotherapy, further contribute to this inflammatory milieu [17]. Collectively, these signals enhance APC licensing, promote CTL and natural killer (NK) cell activity, and support effective anti-tumor immune responses.

Despite this rapidly expanding body of literature, a critical conceptual challenge remains. In practice, ICD is frequently inferred from the detection of surrogate biomarkers such as DAMP exposure or cytokine release and is often simplified in vitro systems [18]. However, the mere presence of immunostimulatory signals does not necessarily equate to the induction of a productive adaptive immune response [19]. Indeed, inflammatory cell death, DAMP emission, and immune cell recruitment can occur without resulting in tumor-specific T cell priming or immunological memory [20]. From a functional standpoint, a feature is the ability to elicit protective, antigen-specific immunity, a property most rigorously demonstrated by vaccination assays and immune-dependent tumor rejection in vivo [21,22].

This distinction is particularly relevant in the context of the tumor microenvironment, which can actively suppress or distort immune response through metabolic constraints, inhibitory cytokines, dysfunctional APCs, and chronic inflammation [22,23]. As a result, many cell death modalities that appear immunogenic in a reductionist setting may fail to translate into effective anti-tumor immunity in vivo [24]. The widespread and often uncritical use of the term ICD has therefore led to conceptual ambiguity, reduced reproducibility, and difficulties in comparing results across studies [25,26].

**Figure 1:**
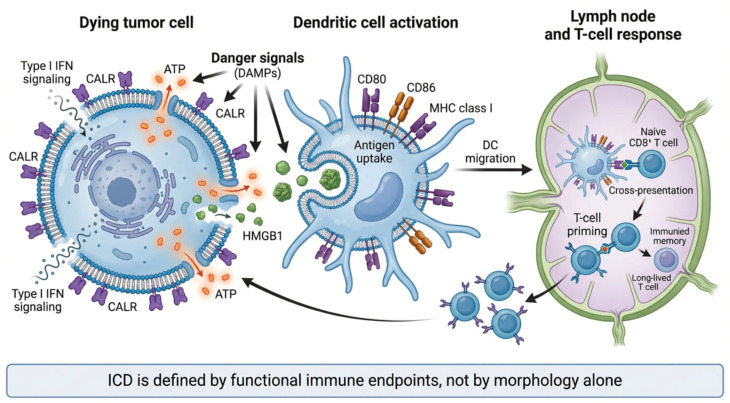
Conceptual framework of immunogenic cell death (ICD) and adaptive immune priming. Therapy-induced regulated cell death can become immunogenic when tumor cells emit coordinated danger signals, including pre-mortem calreticulin (CALR) exposure and the release of ATP and HMGB1, together with type I interferon signaling. These cues promote dendritic-cell (DC) recruitment, antigen uptake, maturation, and cross-presentation, leading to priming of tumor-specific CD8+ T cells and the establishment of durable antitumor immunity. ICD should be concluded from functional immune endpoints rather than morphology alone.

In this review, we argue that ICD should be defined and evaluated primarily as a functional immunological outcome, rather than as a collection of molecular surrogates (Figure 1). We propose an evidence-based framework to distinguish true ICD from DAMP-associated or inflammatory cell death, enhancing common experimental pitfalls, and discuss emerging gray zones, including senescence-associated phenotypes and non-lethal stress responses, that further blur current definitions. By establishing minimal experimental standards and grading levels of evidence, we aim to provide a practical guide for investigators and reviewers seeking to rigorously assess ICD and its relevance for cancer immunotherapy.

We would like to emphasize that we are not proposing a new definition of immunogenic cell death that departs from existing consensus guidelines. Rather, we clarify and reinforce the consensus view, particularly the requirement for functional, antigen-specific adaptive immunity and the vaccination-rechallenge paradigm as the gold standard, and we operationalize it into an evidence-graded framework that links the strength of an ICD claim to the depth of immunological validation.

ICD is not a single cell-death pathway; rather, it is a functional state that can be achieved through distinct regulated cell-death modalities in a context-dependent manner. For clarity, we summarize below the major cell-death modalities that have been reported to elicit ICD and briefly outline their defining features (Box 1).

Box 1 **Regulated cell death modalities are most commonly reported to elicit immunogenic cell death (ICD) in cancer models.**

**Immunogenic apoptosis is** a regulated apoptotic program that, under specific stress conditions, coordinates pre-mortem danger signalling, such as calreticulin (CALR) exposure and post-mortem release of ATP and HMGB1, in a spatiotemporally ordered manner that supports efficient dendritic-cell uptake and cross-priming [27]. Thus, immunogenic apoptosis should not be equated with apoptosis per se, since apoptotic death is often immunologically silent; rather, it refers to a specific apoptotic context in which cell death is coupled to productive adaptive immune activation [28].

**Necroptosis is** a regulated lytic cell-death modality driven by RIPK1/RIPK3/MLKL signalling. Because it promotes membrane rupture, necroptosis can favor antigen release and innate sensing [29]. However, as with other ICD-competent modalities, immunogenicity cannot be inferred solely from DAMP release or inflammatory features and must be supported by evidence of antigen presentation and antigen-specific T-cell priming [30].

**Pyroptosis is** a regulated lytic-cell death modality mediated by gasdermins, typically associated with inflammasome activation and IL-1 family cytokine release [31]. Although pyroptosis is frequently highly inflammatory and DAMP-rich, it should be considered ICD only when it leads to productive antigen-specific adaptive immune responses [32].

**Ferroptosis is** an iron-dependent cell-death modality driven by lipid peroxidation [33]. Depending on the inducer, treatment intensity, and microenvironmental context, ferroptosis may either promote immunostimulatory signaling or generate oxidized lipid mediators with immunosuppressive effects. Its classification as ICD is therefore strongly context dependent [34].

**Cuproptosis is** a recently characterized regulated-cell death modality caused by copper-dependent proteotoxic stress linked to lipoylated mitochondrial proteins [35]. Its possible immunogenic properties are of growing interest, but in our review, the available evidence remains preliminary. At present, cuproptosis should be regarded as an emerging RCD modality with potential ICD relevance rather than an established ICD pathway.

**Other regulated modalities with reported ICD potential** include mitotic catastrophe-associated death and therapy-induced senescence followed by immune-mediated clearance (“ICD-adjacent” contexts) [36,37]. Such scenarios may display DAMP release or inflammatory hallmarks, but they should only be designated as ICD when they demonstrably induce adaptive immunity, ideally including immunological memory.

Across these modalities, ICD is not assigned by morphology or DAMP release alone; it is concluded from functional immune endpoints, particularly antigen-specific priming, and, ideally, protective immunity in immunocompetent settings.

## 2. From Danger Signals to Functional Immunity: What Truly Defines ICD

A central misconception in the ICD field is to treat the emission or surface exposure of a few canonical danger signals as a direct proxy for “immunogenicity” [38]. In reality, ICD is defined by its immunological consequence, the induction of a tumor-antigen-specific adaptive immune response, and, ideally, long-term protective memory, rather than by any single molecular event occurring in dying cells [39]. The distinction is not semantic: it determines how we interpret biomarkers, how we design experiments, and how we decide whether a therapy is truly “immunogenic” in vivo [40]. This shift in thinking is explicitly supported by conceptual and methodological frameworks proposing that ICD cannot be inferred from morphology or from isolated biochemical hallmarks alone but must be validated functionally (ideally in vivo) [41].

### 2.1. Three Related, but Different, Layers: Molecular Immunogenicity, Inflammation, and Adaptive Immunity

ICD-related processes can be organized into three related layers:

- Molecular immunogenicity (danger signals by dying cells): This refers to the capacity of stressed/dying tumor cells to emit or expose immunostimulatory cues such as calreticulin (CALR) exposure, ATP secretion, HMGB1 release, and additional signals, including type I interferon-linked programs and chemokines (e.g., CXCL10) or other “find-me/activate-me” mediators [42,43]. In some studies, multiple intracellular stress modules (e.g., ER stress/UPR, autophagy, inflammasome signaling, nucleic acid sensing via PRRs) converge on distinct danger outputs that support antigen presentation and T cell priming [44,45,46].
- Local inflammation (innate activation in the tissue): Inflammation is often present during tumor cell killing, but inflammation alone does not guarantee antigen-specific immunity [47,48]. A treatment can provoke cytokines or innate recruitment yet fail to generate effective cross-priming, especially if antigen capture/presentation is inefficient or if suppressive circuits dominate [49,50].
- Antigen-specific adaptive immunity (the ICD endpoint): here, the operational endpoint is the generation of tumor-specific T cells (especially CD8+ T cells) that control rechallenge and support durable memory [51,52]. This is why the “gold standard” for bona fide ICD is not a molecular readout, but a vaccination-rechallenge experiment in immunocompetent syngeneic mice [53].

These layers explain why a biomarker panel can look “ICD-like” while the immune outcome is absent.

### 2.2. Constitutive Versus Inducible DAMPs: What They Inform, and What They Cannot

A practical way to clarify biomarker meaning is to separate danger signals into the following:

- Constitutive DAMPs (cDAMPs): Pre-existing intracellular molecules that become immunologically “visible” when redistributed or released (e.g., ecto-CALR, extracellular ATP, HMGB1) [54,55]. These can reflect cellular stress and membrane integrity changes and are useful surrogate hallmarks for screening and mechanistic studies [56].
- Inducible DAMPs (iDAMPs): Signals that require active transcriptional or signaling programs (not merely leakage), such as type I IFN-linked inflammatory programs and downstream chemokines (e.g., CXCL10), often connected to PRR signaling and cell-intrinsic responses [57].

However, a critical limitation is that none of these hallmarks, alone or even combined, predict ICD with absolute certainty. Some studies suggest that some conditions produce strong CALR exposure, ATP secretion, and HMGB1 release, yet fail to elicit antigen-specific memory unless additional requirements are met [58].

Methodologically, this problem is also acknowledged in the cancer-ICD assay literature: major ICD biomarkers are difficult to quantify in vivo, ex vivo measurements can be artifact-prone, and therefore functional assays remain essential [53].

- Antigenicity versus adjuvanticity: both are necessary, neither is sufficient alone: To progress beyond a “DAMP checklist”, it is useful to frame ICD as requiring two complementary properties:
- Antigenicity: The availability of antigens (including neoantigens) that can be processed and presented [59].
- Adjuvanticity: The ability of the dying-cell context to provide the right activation signals so that antigen presentation leads to productive priming rather than tolerance [60].

The detection-focused review emphasizes that ICD integrates adaptive immune responses and the emission of adjuvant-like signals (DAMPs) that enable that transition [61]. Yet various studies make clear that the field has moved away from assuming that morphology or biochemistry automatically maps to immunogenicity; instead, ICD is defined by whether antigenicity and adjuvanticity successfully converge into protective immunity [62].

Finally, multiple types of ICD exist, each relying on partly distinct DAMP/mediator panels [63]. This explains why one tumor/therapy combination may display some hallmarks but still fail to reach the immunological endpoint.

### 2.3. The Indispensable Role of Functional APCs and CD8+ T Cells

Even a particularly “dangerous” dying tumor cell cannot generate immunity in isolation. ICD is an ecosystem event requiring the following:

Functional antigen-presenting cells (APCs), especially dendritic cells (DCs): the mechanistic model of chemotherapy-driven ICD explicitly links danger signals to DC recruitment, antigen uptake, maturation, and cross-presentation, culminating in T cell priming [64,65].

Effective CD8+ T cell priming, expansion, and memory formation: In the vaccination-rechallenge paradigm, protection is measured by tumor-free survival after rechallenging, which is a direct readout of adaptive immunity and memory [66,67].

This requirement also explains why immunodeficient settings can mask DC: if the antitumor effect depends largely on adaptive immunity, then eliminating immune competence will compromise or abrogate the apparent benefit [68].

### 2.4. How to Prove ICD (and Why the “Golden Standard” Matters)

Positioning relative to prior consensus documents is important. Our framework is intended as an operational extension of the existing consensus (e.g., the KITC guidelines), not as a competing redefinition: the defining endpoint remains antigen-specific adaptive immunity, ideally with protective memory, and vaccination-rechallenge in immunocompetent models remains the highest-confidence experimental demonstration. The novelty here is the explicit hierarchy-of-evidence logic, adapted from evidence-based medicine, to guide what can (and cannot) be claimed from common surrogate assays and intermediate functional readouts.

Because surrogate biomarkers can mislead, the field converges on a hierarchy of validation:

- Surrogate/screening-level evidence: Measurement of hallmarks DAMPs (CALR exposure, ATP secretion, HMGB1 release) and related pathways; these are useful for discovery pipelines and mechanistic dissection. The detection review discusses method development and screening logic for ICD biomarkers and inducers [39,53,69].
- Intermediate functional evidence: DC maturation/activation assays and readouts that connect dying-cell signals to APC function (helpful especially when human systems limit in vivo testing) [39,53,69].
- Definitive evidence (bona fide ICD): Vaccination-rechallenge in immunocompetent syngeneic mice. The protocol chapter lays out this approach explicitly: vaccination with treated tumor cells followed by rechallenge with viable tumor cells of the same type [39,53,69].

Importantly, this protocol is also presented as the in vivo “gold standard” because alternative readouts can be confounded by off-target immunostimulation or immunoregulatory effects unrelated to tumor-cell ICD per se.

**Figure 2:**
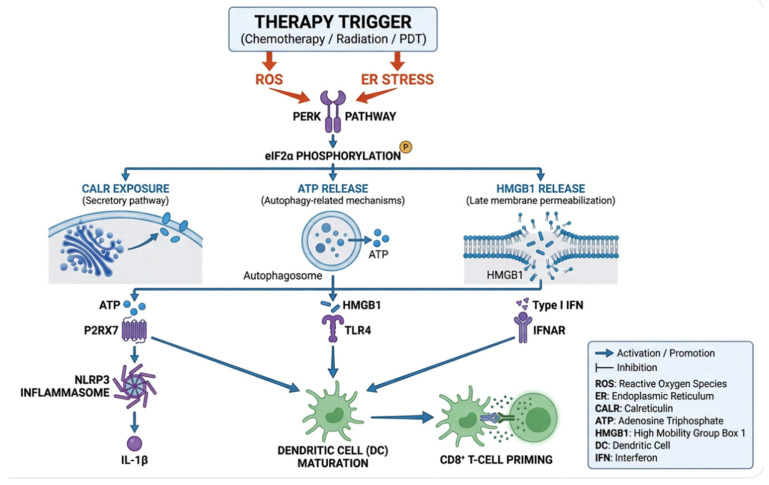
Stress signaling pathways connecting therapy exposure to canonical ICD hallmarks. Anticancer therapies, including but not limited to chemotherapy, radiotherapy, and photodynamic therapy (PDT), can induce reactive oxygen species (ROS) and endoplasmic reticulum (ER) stress, engaging PERK-eIF2α signaling and downstream processes that drive CARL exposure, ATP secretion (often coupled to autophagy-related mechanisms), and HMGB1 release at later stages. ATP-P2RX7 and HMGB1-TLR4 sensing, together with type I interferon signaling, converge on dendritic cell maturation and efficient antigen cross-presentation, ultimately enabling tumor-specific T-cell priming.

Also, DAMP emission should be interpreted as evidence of immunogenic potential rather than proof of ICD, which is ultimately defined by antigen-specific adaptive immunity. This conceptual separation is summarized in Figure 2.

Finally, the existence of consensus guidelines for ICD detection underscores how the community has tried to standardize surrogate hallmarks and workflows, while still recognizing that functional validation is indispensable.

Taken together, the considerations discussed above underscore a simple but often overlooked point: the immunological consequences of tumor cell death cannot be inferred solely from the presence of danger signals or inflammatory cues. Although constitutive and inducible mediators released by dying cells provide important information about cellular stress and immune engagement, their detection does not necessarily imply that tumor antigens have been effectively processed and translated into a durable, antigen-specific immune response. The decisive steps that confer immunogenicity occur downstream, at the level of antigen presentation and T-cell activation, and are highly dependent on immune competence and context.

Recognizing this distinction is necessary for interpreting experimental results and for avoiding overextension of the term immunogenic cell death. The question, therefore, shifts from which signals are present to what immune outcome is ultimately achieved. In the following section, we build on this conceptual separation to examine how different types of experimental evidence support, or fail to support, claims of immunogenic cell death, and to delineate the criteria that distinguish molecular correlates from functionally meaningful immune responses.

## 3. Experimental Evidence of Immunogenic Cell Death: A Hierarchy of Validation

The increasing use of the term ICD across experimental oncology has enhanced the need for a clear distinction between surrogate molecular readouts and functionally meaningful immune outcomes [70,71,72]. As discussed in the previous section, the emission of danger signals and the induction of inflammation do not, by themselves, establish that tumor cell death has resulted in antigen-specific adaptive immunity. To address this gap, reflecting progressively stronger levels of immunological validation. This hierarchy does not imply that lower levels are uninformative, but rather that different types of evidence support different strengths of ICD claims [73].

### 3.1. Level 0—Correlative Evidence: Molecular Hallmark of Danger

The most accessible and widely reported form of evidence for ICD is the detection of molecular hallmarks associated with danger signaling [72]. These include the surface exposure of calreticulin, extracellular ATP, and HMGB1, induction of endoplasmic reticulum (ER) stress responses (such as phosphorylation of eIF2α), and production of inflammatory cytokines or chemokines, including type I interferon-related signatures [15]. Such readouts provide important mechanistic insight into how dying cells may acquire immunostimulatory properties and are particularly useful for screening candidate ICD inducers or dissecting stress pathways [39].

However, multiple consensus documents and experimental studies emphasize that these markers are correlative rather than definitive [15,39,72]. Hallmark DAMPs can be detected in contexts that fail to generate tumor-specific adaptive immunity, and their presence does not predict immunogenicity with absolute certainty [72]. Accordingly, Level 0 evidence should be interpreted as indicating potential immunogenicity, not ICD itself. Claims based exclusively on this level of risk conflate cellular stress or damage with immune instruction.

### 3.2. Level 1—Innate Immune Activation: Functional Perception of Danger

A higher level of support for ICD is provided when molecular danger signals are shown to be functionally perceived by innate immune cells, particularly APCs [74,75]. This level includes evidence of dendritic cell recruitment, maturation, cytokine production, and the capacity of APCs to uptake material from dying tumor cells. In vitro co-culture systems, assessment of costimulatory molecule upregulation, and assays measuring antigen cross-presentation fall within this category [76,77,78].

Level 1 evidence indicates that danger signals are not merely present but are biologically active, initiating immune-relevant processes [79]. Nonetheless, innate activation alone does not guarantee productive adaptive immunity [80]. APC activation can occur without leading to effective T-cell priming, for example, when antigen processing is inefficient or when suppressive cues dominate [81]. Thus, Level 1 evidence supports immune engagement, but still falls short of demonstrating ICD as an immunological outcome.

### 3.3. Level 2—Adaptive Immune Priming: Antigen-Specific T-Cell Responses

The transition from innate immune activation to antigen-specific adaptive immunity represents a critical threshold in ICD validation [82]. Level 2 evidence is defined by the demonstration that tumor cell death leads to the priming of tumor-specific T cells, most commonly CD8+ cytotoxic T lymphocytes. Experimental indicators include antigen-specific T-cell activation, proliferation, cytokine production, and dependence on major histocompatibility complex class I (MHC-I)-mediated antigen presentation [83,84,85].

At this level, ICD begins to acquire a functional immunological meaning. Importantly, Level 2 evidence distinguishes ICD from inflammatory or bystander immune responses by showing that immune activation is antigen-directed [84]. Nevertheless, adaptive priming does not necessarily equate to durable protection. T-cell responses may be transient, quantitatively insufficient, or functionally impaired, enhancing the need for further validation when claiming bona fide ICD [83,85].

Operationally, we consider Level 2 evidence sufficient to claim ICD-associated antigen-specific adaptive priming (e.g., and ICD-relevant functional immune outcome) in mechanistic studies, provided antigen specificity and immune-context requirements (e.g., MHC-I dependence) are demonstrated. However, we recommend reserving the term “bona fide ICD” for level 3 validation, because protection and memory cannot be inferred from priming alone.

### 3.4. Level 3—Protective Immunity: In Vivo Validation of ICD

The most stringent and widely accepted evidence for ICD is the demonstration that dying tumor cell function as an endogenous vaccine, capable of inducing protective, long-lasting immunity [86]. This is classically assessed using vaccination, rechallenge experiments in immunocompetent syngeneic mouse models [53]. In this setting, animals vaccinated with tumor cells undergoing immunogenic death are protected against subsequent challenge with viable tumor cells of the same type.

Level 3 evidence directly tests the defining feature of ICD: the induction of immunological memory. Additional refinements, such as immune cell depletion or genetic ablation of key immune pathways, further strengthen conclusions by demonstrating dependence on specific immune components (e.g., CD8+ T cells or dendritic cell subsets) [87,88]. Because this level integrates all upstream requirements, danger signaling, innate activation, antigen presentation, and adaptive immunity, it is regarded as the gold standard for establishing bona fide ICD.

In this evidence-grading scheme, Level 3 constitutes the threshold for bona fide ICD, because it directly demonstrates vaccine-like protection and immunological memory in immunocompetent settings and can be further strengthened by immune-dependence testing.

### 3.5. Level 4—Translational Relevance: Functional Correlates in Human Settings

The final level of evidence addresses the relevance of ICD-related mechanisms in human disease. This includes correlations between ICD-associated pathways and clinical outcomes, such as links between polymorphisms in danger-sensing receptors, immune infiltrates, or expression of ICD-related markers and therapeutic efficacy [89]. Evidence at this level can provide important support for the clinical significance of ICD mechanisms and guide biomarker development [90].

However, translational correlations alone cannot substitute for functional validation. Human studies are inherently constrained by ethical and technical limitations and rarely allow direct testing of causality [91,92]. Consequently, Level 4 evidence should be interpreted as contextual and supportive, reinforcing, but not redefining, ICD claims established at higher functional levels.

Taken together, the evidence summarized in this section highlights that not all experimental observations support the same strength of conclusions regarding immunogenic cell death. Molecular hallmarks and innate immune activation provide valuable insight into cellular stress responses and immune engagement, but only higher levels of functional validation establish that tumor cell death has resulted in antigen-specific, protective immunity. Distinguishing between these layers of evidence is necessary for the consistent interpretation of experimental findings and for avoiding overgeneralization of ICD claims. The different categories of experimental support and their respective strengths and limitations are summarized in Table 1.

This graded view of experimental evidence also helps explain why apparently similar forms of tumor cell death can lead to divergent immune outcomes depending on context. In the following section, we examine how features of the tumor microenvironment and immune competence influence whether immunogenic signals are effectively translated into adaptive anti-tumor immunity.

## 4. What ICD Is Not: Common Misinterpretations and Experimental Pitfalls

The rapid expansion of ICD-related literature has been accompanied by parallel infiltration of ICD claims, often by overinterpretation of surrogate biomarkers or by experimental [39]. Designs that cannot discriminate between danger signaling, inflammation, and bona fide antigen-specific immunity [93]. Multiple consensus and methodological papers stress that ICD should not be concluded from single-layer observations, and that rigorous interpretation requires appropriate immune-context controls [94,95,96].

### 4.1. Acute Inflammation Is Not Equivalent to ICD

Acute inflammation, cytokine release, or transient activation of innate pathways can occur in response to many forms of tissue damage and tumor cell death [97]. However, inflammation can remain non-productive or even become immunosuppressive depending on timing, myeloid polarization, antigen presentation quality, and the tumor microenvironment [98]. Consensus guidelines emphasize that inflammatory readouts cannot substitute for evidence of adaptive, antigen-specific immunity [99].

Reports that end at “increased inflammatory cytokines” or “higher immune infiltration” should be interpreted as innate engagement unless accompanied by evidence of tumor antigen-specific T-cell priming and immune dependence [100,101].

### 4.2. Therapeutic Benefit Is Not Proof in ICD

Tumor control after treatment can reflect multiple mechanisms that are unrelated to ICD (direct cytotoxicity, anti-angiogenic effects, stromal disruption, or immune activation independent of tumor cell death) [102,103]. Methodological papers caution that some interventions may stimulate immune cells directly, creating an illusion of ICD even if the dying tumor cells are not acting as an endogenous vaccine [69,104].

Any claim that “therapy X induces ICD because it improves tumor control” requires immune-dependence testing (e.g., CD8+ depletion, Batf3 dependence, MHC-I dependence) and ideally a vaccination-rechallenge demonstration if the claim is bona fide ICD.

### 4.3. Massive DAMP Release from Necrosis Does Not Guarantee Adaptive Immunity

DAMPs are central to ICD biology, but consensus documents and reviews repeatedly underscore that DAMP emission is not sufficient to conclude ICD [105]. Necrotic damage can yield abundant extracellular DAMPs and inflammation yet fail to generate protective immunity if antigen processing and cross-presentation are inefficient, if APCs are dysfunctional, or if T-cell priming is blocked by suppressive circuits [106,107].

A key point of an important study is that ICD is not a single stereotyped event but can occur via distinct variants with different signal combinations, and no single hallmark panel predicts immunogenicity with certainty across contexts [108].

“More DAMPs” is not a linear proxy for “more ICD”. DAMP data must be interpreted as adjunctivity-related correlates whose meaning depends on downstream immune integration [74].

### 4.4. In Vitro-Only Systems Cannot Establish ICD

High-content and high-throughput assays are valuable for screening ICD-associated hallmarks, but the 2014 detection guideline explicitly notes that the gold-standard evaluation relies on immunocompetent murine vaccination experiments, which is an approach incompatible with large screens, hence the use of surrogates in vitro [39].

Without an intact system, one can only claim “ICD-like hallmarks” or “danger signaling”, not bona fide ICD, unless the study includes robust functional bridging evidence that reaches adaptive priming (e.g., antigen-specific T-cell assays) and immune dependence.

### 4.5. Missing Immune-Context Controls: The Most Frequent Source of False Positives

Across consensus and methodological documents, a recurring theme is that claims of ICD often omit the controls required to demonstrate immune causality [109]. Examples include the following:

- MHC-I dependence: To show that antigen presentation is required for the adaptive effect [110];
- CD8+ T cell dependence: Via depletion or genetic models, to establish adaptive effector necessity [111];
- Batf3-dependent DCs/cross-presenting APCs: To demonstrate the APC bottleneck [112];

Innate sensing nodes (e.g., STING/type I IFN axis): When claims rely on nucleic-acid sensing or IFN-driven recruitment/priming [113].

The absence of these controls should downgrade an ICD claim to a lower delivery (e.g., danger signaling or innate activation), even if hallmark DAMPs are robust [12].

Altogether, these examples show that any reported “ICD” findings are better interpreted as evidence of danger signaling of inflammation unless antigen-specific adaptive immunity and immune dependence are directly demonstrated [114]. Aligning conclusions with the depth of validation and incorporating immune-context controls where appropriate helps prevent false-positive claims and improves comparability across studies. The most common misinterpretations, alongside practical corrective controls and suggested wording, are summarized in Table 2.

This discussion also enhances an important reality: even when experimental design is rigorous, immune outcomes remain highly context-dependent. In the next section, we examine how factors such as the tumor microenvironment, APC functionality, and immune competence determine whether immunogenic signals are successfully translated into adaptive anti-tumor immunity.

## 5. Context Dependency of ICD: Why the Same Death Is Not Always Immunogenic

Even when the tumor cells display robust ICD-associated hallmarks, the resulting immune outcome can vary widely across tumor types, models, and patients, because the steps that define bona fide ICD occur downstream of the dying cell, within the tumor immune ecosystem [115]. In practice, the conversion of danger signals into antigen-specific adaptive immunity depends on whether antigen-presenting circuits remain functional, whether innate licensing signals reach the “right” cellular compartment, and whether biochemical and metabolic conditions allow immune cells to execute cross-presentation and effective priming rather than drift toward tolerance or exhaustion [116,117]. This contextual view is consistent with the idea that DCs orchestrate T cell immunity at multiple sites, tumor-draining lymph nodes, and tumors themselves, making DC state and positioning a decisive bottleneck for whether immunogenic signals become durable T cell memory [118,119]. To maintain conceptual focus, this section does not aim to review tumor immunology broadly; instead, we enhance only those context variables that directly cofound ICD attribution by uncoupling ICD-associated danger signaling from antigen-specific priming and protective immunity.

### 5.1. The Dendritic-Cell Bottleneck: Antigen Capture and Cross-Presentation as a Limited Step

A recurring reason why “ICD-like” molecular signature does not translate into antigen-specific immunity is that tumor antigens and danger signals must be processed through a cellular bottleneck dominated by DCs [120]. DCs coordinate two major checkpoints of the cancer-immunity cycle: priming of naïve T cells in tumor-draining lymph nodes and support of effector T-cell function at the tumor site [121]. In this view, the immunogenic potential of dying tumor cells is only realized if DCs are present in sufficient numbers, are appropriately activated, can acquire tumor material, and can cross-present antigenic peptides to CD8+ T cells [122]. Reviews that focus on DC biology in cancer emphasize that intratumoral DC scarcity, altered maturation states, and expression of immunoregulatory programs can limit effective T-cell responses, even when the antigen is available [123,124]. This helps explain why studies may detect robust DAMP emission and inflammatory changes yet fail to see strong tumor-specific CD8+ priming: the limiting step is not necessarily the presence of danger signals, but whether cross-presenting DC programs can execute the “translation” of those signals into adaptive immunity [125,126]. Mechanistically, impaired DC trafficking from tumor to lymph node, reduced cross-presentation capacity, or diversion toward tolerogenic antigen presentation can each uncouple dying-cell signals from protective immunity [127]. From a conceptual standpoint, that bottleneck aligns with the definition of ICD as an outcome: without functional DC-mediated cross-presentation and subsequent CD8+ T-cell priming, the process stalls upstream of the endpoint, yielding local inflammation without durable, antigen-specific memory [128,129].

### 5.2. Innate Licensing Is Compartmental: How IFN-STING Signaling Conditions Priming

Beyond antigen handling, the conversion of tumor cells into effective adaptive immunity often depends on innate “licensing” programs that operate in specific cellular compartments, particularly antigen-presenting cells [130,131]. Type I interferon-linked responses are frequently described as critical for establishing a priming-permissive environment, and the cGAS-STING axis is a major upstream driver connecting cytosolic DNA sensing to interferon and inflammatory gene programs [132,133]. Importantly, the magnitude and quality of these outputs can be cell-type dependent (e. g., differing consequences when the pathway is engaged in tumor cells versus myeloid/APC compartments), and the downstream balance of transcriptional programs can vary across contexts [134]. This provides a mechanistic rationale for divergent outcomes when upstream dying-cell features appear similar: if innate licensing programs are insufficient, spatially misaligned, or actively suppressed by the tumor microenvironment, danger signaling may remain confined to local innate activation without progressing to robust priming [135]. In addition, tumors can impose constraints on interferon responsiveness, myeloid activation states, and APC function, thereby lowering the probability that antigen presentation culminates in productive CD8+ activation [136]. As a result, IFN-STING activity should be interpreted as a context-sensitive contributor to the ICD cascade rather than a universal “stamp” of immunogenicity: the same treated tumor cells can lead to different adaptive immune outcomes depending on whether licensing signals effectively support cross-presentation and priming in the relevant APC populations [137,138].

### 5.3. The ATP-to-Adenosine Switch: A Biochemical Short-Circuit of Adjuvanticity

Extracellular ATP is commonly discussed as an immunostimulatory signal emitted by stressed or dying tumor cells, but its functional meaning depends on how long it persists and how it is metabolized in situ [139]. In many tumors, ATP is rapidly hydrolysed through ectonucleotidases, classically CD39 (ENTPD1) and CD73 (NT5E), leading to adenosine accumulation [140]. Reviews of purinergic regulation in cancer describe this axis as a major immunoregulatory circuit that can transform a potentially activating extracellular nucleotide milieu into an adenosine-dominated suppressive environment [141]. This conversion is conceptually important for ICD interpretation: a therapy may trigger ATP release (a Level 0 hallmark), yet the local biochemical context may remove ATP rapidly and replace it with adenosine signaling that suppresses immune effector functions [142]. The consequence is an apparent paradox, strong danger-signal emission with limited adaptive immunity, which can be resolved by viewing adjuvanticity as “fragile” and environmentally constrained [143]. In practical terms, ATP-associated immunogenic potential is therefore contingent upon the balance of nucleotide release versus nucleotide breakdown, the spatial distribution of CD39/CD73 activity, and the relative dominance of purinergic activation versus adenosinergic suppression within the tumor microenvironment [144]. This reinforces the broader argument: ICD is not a fixed molecular phenotype, but an immune outcome that requires permissive biochemical conditions to sustain activating cues long enough for APC integration and T-cell priming [145].

### 5.4. Hypoxia and Metabolic Stress: When Immune Cells Cannot Execute the Program

Even when ICD-associated danger cues are present, physicochemical constraints within the tumor microenvironment can prevent their translation into antigen-specific adaptive immunity [146]. Hypoxia and nutrient competition can reduce antigen presentation (including MHC-I availability) [147] and impair dendritic-cell cross-presentation and T-cell effector fitness, thereby lowering the probability that priming and durable memory will occur [148,149]. From an ICD-interpretation standpoint, these constraints explain why “ICD-like” hallmarks may coexist with limited adaptive endpoints: the bottleneck is immune execution capacity downstream of the dying cell rather than danger-signal emission per se [150,151]. Together, these findings provide a mechanistic basis for why ICD-associated signals may fail to yield adaptive immunity: hypoxia can reduce antigen presentation; metabolic stress can compromise APC and T-cell functionality; and nutrient limitation can skew immune programs away from durable effector responses.

### 5.5. Practical Consequence: Interpret ICD as a Conversion Problem, Not a Marker Problem

These context layers converge on a pragmatic interpretation that aligns with the paper’s core message [152]. ICD-associated markers describe an upstream potential, danger cues, and stress-associated signaling emitted during tumor cell death, but whether that potential becomes antigen-specific adaptive immunity depends on conversion efficiency across several bottlenecks [153]. The most consistent limiting steps include (i) DC availability, positioning, and cross-presentation capacity [154]; (ii) innate licensing programs that support priming in antigen-presenting compartments [155]; (iii) the biochemical fate of key mediators such as extracellular ATP in nucleotide-rich, enzyme-regulated tumor spaces [156], and (iv) the physicochemical/metabolic environment that constrains antigen presentation and immune-cell fitness [157]. Viewed through this lens, divergent outcomes across tumors become more interpretable: similar dying-cell phenotypes can lead to distinct immune trajectories because tumors differ in DC competence and across-presentation, in the balance of purigenic versus adenosinergic signaling, and in hypoxia/metabolic stress that reshape MHC-I presentation and T-cell function [158,159]. This contextual framing also helps keep ICD claims appropriately bounded: the presence of DAMPS or inflammatory mediators remains informative, but conclusions about ICD must ultimately be supported by evidence that the tumor environment allowed these signals to be translated into CD8+ priming and protective immunity [10,160].

## 6. Gray Zones in ICD: When Immunogenic Signals Do Not Map Clearly onto “Immunogenic Cell Death”

A growing body of work suggests that immunogenicity in cancer therapy often emerges along a continuum rather than as a binary property of a single death pathway [161]. This creates several “gray zones” where tumors exhibit ICD-associated signals (or immune-stimulatory consequences) without meeting the strict functional definition of ICD as antigen-specific protective immunity [162,163]. These situations are not marginal; they explain much of the heterogeneity in the literature and why readers can encounter apparently contradictory conclusions across models [164]. Methodological and consensus-oriented papers repeatedly emphasize that hallmark measurements (e.g., CALR exposure, ATP/HMGB1 release, interferon-linked signatures) provide valuable information about danger signaling and adjuvanticity, but cannot, on their own, settle whether the endpoint has been reached [165,166].

### 6.1. Immunogenic Modulation Versus ICD: Survival with Heightened Immune Visibility

One of the most important gray zones is immunogenic modulation, in which sublethal stress or therapy exposure does not kill all tumor cells but alters surviving cells in ways that make them more vulnerable to immune attack [167]. This concept has been described as a continuum spanning from immune-sensitizing phenotypic changes to bona fide ICD, particularly in the radiotherapy setting. In practice, immunogenic modulation can include increased antigen presentation machinery, stress–ligand display, or changes in susceptibility to cytotoxic T lymphocytes (CTLs), sometimes accompanied by CALR exposure that is linked to endoplasmic reticulum stress [168]. The interpretive pitfall is straightforward: studies may report CALR exposure or enhanced CTL killing after irradiation/chemotherapy and label this as ICD, when the dominant biology may instead be “tumor cells survive, but become easier targets” [168]. This distinction matters because the downstream therapeutic strategy differs; immunogenic modulation points toward rational combinations that exploit immune sensitization (e.g., checkpoint blockade or adaptive T-cell approaches), whereas ICD claims imply that dying cells themselves are acting as an endogenous vaccine [169,170,171].

### 6.2. Pre-Lethal and “Particular ICD” Signatures: Danger Signals Without the Full Immune Cascade

A second gray zone concerns pre-apoptotic or incomplete ICD-like signatures, where key hallmarks appear but the system does not progress to durable, antigen-specific immunity [172]. Classic mechanistic work established that CALR exposure can occur early and is functionally important for phagocytic uptake and immunogenicity in some settings [173]. However, later syntheses stress that hallmark combinations are not universally predictive and that DAMPs represent only one segment of a multi-step process [174]. This creates a frequent scenario in practice: tumor cells display surface CALR and release ATP/HMBG1, yet antigen presentation, T-cell priming, or memory formation is weak, because the APC bottleneck is not met, interferon-linked licensing is absent, or the tumor microenvironment neutralizes adjuvanticity [175,176]. A related nuance is active immune escape by “DAMP suppression”, exemplified by reports of tumor-intrinsic programs that prevent CALR exposure and thereby reduce uptake by antigen-presenting cells, and an explicit reminder that “hallmark absence” can be regulated rather than incidental [177,178]. Framing the cases as “partial ICD signatures” (rather than ICD) keeps conclusions aligned with what is actually demonstrated and avoids overextension of biomarker-only claims.

### 6.3. Senescence, SASP, and Immune Activation: Immunogenic Outcomes Without Classical Cell Death

Therapy-induced senescence and related senescent states represent a major gray zone because they can generate strong immune–modulatory signals, sometimes including enhanced antigen presentation and cytokine/chemokine production, without immediate cell death [179]. Recent reviews emphasize that senescence can create distinct immunological vulnerabilities while also producing pro-tumorigenic effects depending on timing and context [180]. Mechanistic and translational studies further suggest that senescent tumor cells may upregulate antigen presentation machinery and become unusually responsive to microenvironmental interferon cues, shaping immune recognition in ways that can promote immune-mediated clearance [181]. At the same time, the senescence-associated secretory phenotype (SASP) is heterogeneous and can support either immune recruitment or chronic immunosuppression and tissue remodeling; chromatin regulation and innate sensing pathways (including cGAS-STING-linked signals) have been implicated in shaping SASP programs and their immune consequences [182,183]. These data complicate simplistic narratives: a therapy can elicit immune activation and even antigen presentation changes through senescence, yet it would be inaccurate to label this as ICD unless functional evidence demonstrates that senescent cells (or their downstream death) generate antigen-specific protective immunity [184].

### 6.4. Bystander Killing, Payload Diffusion, and “Immunogenic Effects Beyond the Targeted Cell”

Modern therapies can produce immune-relevant outcomes that extend beyond directly targeted tumor cells, creating another interpretative gray zone: bystander killing and broader immune effects that do not necessarily originate from a canonical ICD cascade in the initially hit cells [185]. Antibody–drug conjugates (ADCs), for example, may combine direct cytotoxicity, bystander payload diffusion, and immune-stimulating consequences that resemble ICD in some contexts [186]. These situations matter because they can generate tumor-wide immune remodeling even when only a subset of cells is antigen-positive or directly drug-exposed. The central caution is attribution: immune activation may reflect a composite of mechanisms (bystander death modalities, local inflammation, direct immune stimulation, and antigen release) rather than a clean ICD process [187,188]. Treating these as “ICD-adjacent” effects, then asking what level of functional validation exists, helps preserve the paper’s conceptual rigor while still capturing why such therapies can be clinically compelling [189].

## 7. Practical Standards for Claiming ICD and Translational Benchmarks (with Clinical Examples)

This section provides practical guidance for how to claim immunogenic cell death in a way that matches the strength of the underlying evidence. Building on the distinctions outlined above, we summarize minimal experimental requirements across key biological steps (danger signaling, APC integration, and adaptive immunity), propose translational benchmarks when gold-standard assays are not feasible in humans, and enhance clinically grounded examples where ICD-related mechanisms have been linked to patient outcomes.

### 7.1. Why Is a Stricter Standard Needed?

As ICD has become a widely used concept, the main source of reviewer pushback is not the biological premise, but over-claiming: many studies infer ICD from a subset of hallmarks (e.g., CALR exposure, ATP/HMGB1 release) or from therapeutic benefit, without demonstrating antigen-specific adaptive immunity or immune dependence [190]. Multiple consensus and methodological articles explicitly recommended aligning the strength of the ICD claim with the depth of validation, and emphasize that surrogate markers are informative but not definitive [191].

### 7.2. Minimal Experimental Package for and ICD Claim

For an ICD claim to be robust in an “editorial-grade” sense, reviewers typically look for three elements, each addressing a distinct biological step [192]: (i) danger signaling (what dying cells emit) [42], (ii) immune integration (what APCs do with it) [193], and (iii) adaptive outcome (whether tumor-specific immunity emerges) [194]. The detection-focused guidance stresses that hallmark assays are valuable for discovery and mechanistic work but must be interpreted within a broader immune context [195]. Meanwhile, the KITC consensus document stresses standardized settings, appropriate controls, and clear interpretation rules to avoid labeling partial evidence as ICD [196]. In practical terms, a solid “minimal package” includes measurement of at least two canonical hallmarks (e.g., CALR exposure plus ATP/HMGB1), evidence that APCs can take up dying-cell material and mature (or cross-present antigen), and at least one adaptive readout that demonstrates antigen specificity and dependency (e.g., CD8+ involvement, MHC-I dependence, or antigen-specific assays where feasible) [197,198]. This is also consistent with the classical mechanistic logic of immunogenic chemotherapy, where early CALR exposure supports uptake while additional signals drive DC maturation and optimal MHC-I-restricted presentation to CTLs [199].

### 7.3. Translational Anchors: What “Clinical-Grade” ICD Evidence Looks Like

Because “gold-standard” vaccination-rechallenge experiments are rarely possible in human studies, the most convincing translational evidence usually comes from convergent clinical observations that link ICD-relevant pathways to patient outcomes, ideally with mechanistic plausibility.

The first class of anchors is host-generic associations in pathways required for ICD-associated immune sensing [200]. The HMGB1-TLR4 axis is a canonical example: the interaction of HMGB1 released from dying cells with TLR4 on DCs is linked to cross-priming, and a loss-of-function TLR4 polymorphism has been reported to predict earlier relapse after anthracycline-based chemotherapy in breast cancer cohorts [201,202]. A second example is the ANXA1-FPR1 axis: mechanistic and clinical evidence connect FPR1-mediated DC-dying-cell interactions to chemotherapy-driven antitumor immunity, and a loss-of-function FPR1 SNP has been associated with worse outcomes in chemotherapy-treated patients [203,204]. These studies are particularly valuable to reviewers because they couple an ICD-linked mechanism to a patient-level outcome signal.

A second class of anchors is tumor-tissue correlates, tying ICD-associated markers to immune orientation and prognosis. For instance, analyses in high-grade serous ovarian cancer report that CALR expression/exposure correlates with features of a more active antitumor immune microenvironment and with outcome, supported by retrospective cohorts complemented by functional work on freshly resected samples [205,206]. Importantly, recent syntheses also emphasize that the prognostic meaning of CALR depends on its spatiotemporal distribution rather than expression alone, which provides a nuanced way to discuss biomarker interpretation without overclaiming [207].

A third class of anchors is completed, with therapy-context studies that explicitly evaluate ICD-related biology in standard-of-care regimens. In rectal cancer, neoadjuvant oxaliplatin combined with radiation has been investigated in relation to ICD and tumor-defeating immunity, providing an example of how conventional modalities can be evaluated through an ICD lens in a clinically relevant setting [208,209,210]. In metastatic colorectal cancer, studies have tested whether polymorphisms across the ICD pathway associate with efficacy of oxaliplatin-based chemotherapy, directly building on the premise that oxaliplatin, but not all chemotherapies, can engage immunogenic death programs [208].

### 7.4. Clinical Trial Exemplars Aligned with ICD-Oriented Translational Benchmarks

While formal ICD “vaccination-rechallenge” experiments are not feasible in patients, a growing set of prospective trials and biomarker-driven cohorts can be used to operationalize ICD-oriented benchmarks. In line with an evidence-graded framework, these studies are most informative when they (i) test an ICD-inducing backbone (or a modality with plausible ICD potential), (ii) quantify immune integration within the tumor microenvironment (e.g., antigen-presenting cell activation, T-cell infiltration/activation, interferon programs), and (iii) relate these variables to clinically meaningful endpoints.

Radiotherapy–immunotherapy combinations provide a clinically tractable setting because local tumor damage can be paired with systemic immune activation. In the PEMBRO-RT trial (SBRT followed by pembrolizumab in advanced NSCLC), translational analyses of the tumor microenvironment documented radiation-associated immune remodeling and were designed to probe systemic (abscopal) immune effects, consistent with the concept that therapy-induced tumor damage can license antitumor immunity when immune checkpoints are simultaneously relieved [211].

Gastrointestinal cancers offer additional examples in which ICD-capable backbones are integrated into neoadjuvant platforms together with PD-1 blockade. A randomized phase 2 study in locally advanced rectal cancer evaluated neoadjuvant chemoradiotherapy with or without the PD-1 antibody sintilimab, providing a defined clinical context to relate combined modality treatment to immune and pathological response endpoints [212]. In parallel, total neoadjuvant therapy regimens incorporating PD-1 blockade in high-risk rectal cancer have reported high complete response and organ-preservation rates, reinforcing the translational logic of combining cytotoxic/radiation-induced tumor perturbation with immune checkpoint relief in selected settings [213].

In metastatic colorectal cancer, early-phase studies have prospectively combined pembrolizumab with modified FOLFOX6 (oxaliplatin-containing chemotherapy), incorporating exploratory immune biomarker analyses alongside clinical outcomes. Such designs are directly compatible with an ICD-oriented interpretation because oxaliplatin is a prototypical agent with reported ICD potential in preclinical systems, and the trial structure enables correlative evaluation of immune conversion under a clinically relevant regimen [214].

**Figure 3:**
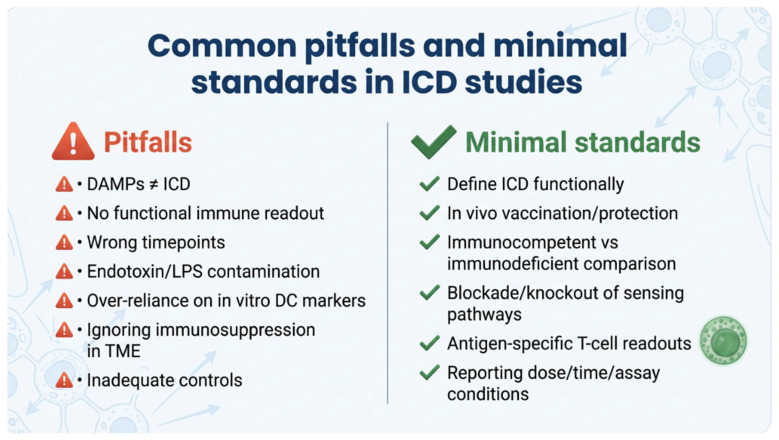
Practical checklist of common pitfalls and minimal experimental standards for ICD attribution. Frequent sources of misclassification include equating individual DAMP measurements with ICD, relying solely on in vitro DC activation markers, and insufficient controls for timing, dose, and contamination. Minimal standards emphasize functional immune validation in immunocompetent settings (ideally vaccination/protection assays), antigen-specific T-cell endpoints, and mechanistic controls that test the requirement of sensing pathways and antigen presentation.

Finally, oncolytic virus-checkpoint inhibitor combinations exemplify strategies that aim to enhance intratumoral antigen release and innate licensing, thereby facilitating adaptive priming. The MSATERKEY-265 study (T.VEC plus pembrolizumab; NCT02263508) provides a large clinical framework for evaluating whether additional intratumoral immune activation translates into improved outcomes, while also illustrating an important caveat: not all biologically plausible “ICD-adjacent” combinations deliver additive clinical benefit, underscoring the need for conservative terminology and benchmark-driven interpretation [108,215] (Figure 3).

## 8. Conclusions

ICD has become a central concept linking tumor cell demise to anti-tumor immunity, yet its broad adoption has also created substantial inconsistency in how the term is used and how evidence is interpreted. Across experimental systems, the presence of ICD-associated hallmarks, such as DAMP exposure, cytokine release, or endoplasmic reticulum stress, should be viewed as indicators of immunogenic potential rather than definitive proof of ICD. The defining criterion remains a functional immune outcome: the efficient conversion of tumor-derived antigenicity and adjuvanticity into antigen-specific adaptive immunity, ideally accompanied by protective memory.

A key message emerging from recent consensus and methodological work is that ICD claims should be proportional to the depth of validation. Surrogate molecular readouts are valuable for discovery and mechanistic insight, but robust conclusions require evidence of immune integration by antigen-presenting cells, demonstrating CD8+ T-cell priming and MHC-I dependence, and, when feasible, in vivo validation through vaccination-rechallenge and immune-dependence testing. Importantly, the frequent divergence between ICD-like molecular signatures and immune outcomes is not paradoxical; it reflects the dominant role of context, including dendritic-cell competence, innate licensing pathways, biochemical constraints such as ATP-to-adenosine conversion, and metabolic or hypoxic stress within the tumor microenvironment.

**Figure 4:**
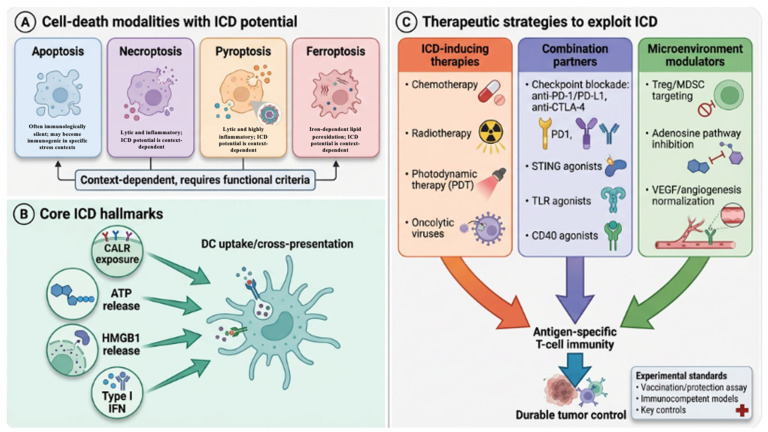
Integrative summary of ICD modalities, hallmarks, and therapeutic strategies to enhance antitumor immunity. (**A**) Multiple regulated cell-death modalities, including immunogenic apoptosis, necroptosis, and ferroptosis, have been reported to elicit ICD in a context-dependent manner. Importantly, none of these modalities should be considered uniformly immunogenic or non-immunogenic solely on the basis of morphology or inflammatory features. (**B**) Canonical ICD hallmarks (CARL exposure, ATP release, HMBG1 release, and type I IFN signaling) promote dendritic cell-mediated antigen uptake and cross-presentation. (**C**) Therapeutic strategies to exploit ICD include ICD-inducing therapies and rational combinations with immunomodulators (e.g., immune checkpoint blockade; STING/TLR/CD40 agonism) and tumor microenvironment interventions (e.g., adenosine pathway inhibition, suppression of Treg/MDSC activity, vascular normalization), collectively reinforcing antigen-specific T-cell immunity and durable tumor control. Key experimental standards required to claim ICD are highlighted.

Looking forward, improving reproducibility and translational impact will require standardized reporting, careful experimental controls, and conservative terminology that distinguishes danger signaling from bona fide ICD. Clinically, the most persuasive evidence will continue to come from convergent observations, which are genetic, immunological, and outcome-based, that map onto mechanistically coherent pathways rather than from isolated biomarker measurements. By treating ICD as an immunological endpoint and adopting evidence-graded standards, the field can better identify truly immunogenic therapies, design rational combinations that overcome contextual barriers, and develop biomarkers that reflect functional immune conversion rather than molecular correlates alone (Figure 4).

## Figures and Tables

**Table 1:** Hierarchy of experimental evidence supporting immunogenic cell death.

Evidence Level	Defining Features	Typical Readouts/Assays	What It Supports	Limitations
Level 0—correlative	Molecular danger signaling	CALR exposure, ATP/HMGB1 release, ER stress markers, cytokines	Presence of immunogenic potential	Does not demonstrate immune function or specificity
Level 1—Innate activation	Functional perception by innate immunity	DC recruitment/maturation, cytokine production, cross-presentation in vitro	Immune engagement by dying cells	Innate activation may not lead to adaptive immunity
Level 2—Adaptive priming	Antigen-specific T-cell responses	CD8^+^ T-cell activation, MHC-I dependency, antigen-specific assays	Initiation of adaptive immunity	Does not ensure durability or protection
Level 3—Protective immunity	Vaccine-like function in vivo	Vaccination-rechallenge, immune depletion studies	Bona fide ICD with memory	Technically demanding, model-dependent
Level 4—Translational relevance	Clinical or human functional correlates	Immune infiltrates, genetic associations, patient outcomes	Human relevance and context	Correlative, rarely causal

**Table 2:** Frequent pitfalls in ICD claims and minimal controls.

Pitfall/Misinterpretation	Why It Misleads	What can be Claimed Instead	Minimal Controls/Upgrades
Acute inflammation ≠ ICD	Inflammation can occur without antigen-specific priming or memory	“Innate activation”/“inflammatory cell death”	Show antigen-specific T-cell priming; test CD8^+^ dependence
Therapeutic benefit ≠ ICD	-Tumor shrinkage can be non-immune or immune-driven independent of tumor-cell vaccine effect	“Therapeutic efficacy with immune engagement”	Separate direct immunostimulation from tumor-cell-death-driven immunity; add immune-dependence tests
Massive DAMPs from necrosis ≠ ICD	DAMP quantity does not ensure APC licensing, cross-presentation, or productive priming	“ICD-like hallmarks”	Add functional readouts and antigen-specific adaptive assays; avoid single-marker conclusions
In vitro-only evidence ≠ ICD	Lacks immune system integration; surrogates are not definitive	“ICD-like hallmarks”	Add functional DC assays and/or in vivo vaccination-rechallenge when feasible
No immune-context controls (MHC-I/CD8/Batf3/STING, etc.)	Cannot establish causality or pathway dependence	“Association with ICD markers”	Add MHC-I dependence, CD8 depletion, Batf3 DC dependence; include innate-sensing dependence when relevant

## Data Availability

No new data were created or analyzed in this study. Data sharing is not applicable to this article.

## Abbreviations

The following abbreviations are used in this manuscript:

RCD	Regulated cell death
ICD	Immunogenic cell death
DAMPs	Damage-associated molecular patterns
APCs	Antigen-presenting cells
DCs	Dendritic cells
CTLs	Cytotoxic T lymphocytes
CALR	Calreticulin
ATP	Adenosine triphosphate
HMGB1	High-mobility group box 1
ADCs	Antibody-drug conjugates
